# Novel optical measurement technique for antimicrobial photodynamic therapy using Scattered Light Integrating Collector (SLIC)

**DOI:** 10.1038/s41598-025-34122-z

**Published:** 2026-01-14

**Authors:** Marianna Leite de Avellar, Hassan Hafeez, Robert Hammond, Ifor D. W. Samuel

**Affiliations:** 1 Division of Infection and Global Health, School of Medicine, University of St Andrews, St Andrews, UK; 2 Organic Semiconductor Centre, SUPA School of Physics & Astronomy, University of St Andrews, St Andrews, UK

**Keywords:** PDT, Antimicrobial resistance, Organic light-emitting diodes, Microbiology, Optics and photonics

## Abstract

Advancing new treatment modalities for microbial infections is a priority, given the threat of antimicrobial resistance. Current work includes the development of new non-traditional treatments, including antimicrobial photodynamic therapy (aPDT). Improvements in the field include innovative technologies that allow screening and optimization of the treatment. In this work it is shown how the Scattered Light Integrating Collector, a novel technology that optically measures bacterial growth in real time, can be used for rapid screening of aPDT using Organic Light Emitting Diodes (OLEDs) as the light source. This technology allows rapid screening of different concentrations of the photosensitizer and real-time observation of the effect of aPDT on *Staphylococcus aureus*. Evaluation of aPDT efficacy using methylene blue and OLEDs as the light source showed significant inhibition of the growth of *S. aureus* (3.06, 3.80, 4.24, 4.98 and 4.84 log_10_ reduction for the concentrations of MB of 1, 2, 4, 8 and 16 µM, respectively). The application of SLIC as a novel technology for evaluation of aPDT is a promising advance that will allow rapid optimization of the treatment.

**Supplementary Information:**

The online version contains supplementary material available at 10.1038/s41598-025-34122-z.

## Introduction

The development of new therapeutic strategies for treatment of infections is a necessity for health care as antimicrobial resistance (AMR) poses a major ongoing threat to public health. The ability of pathogens to evade or overcome the compounds intended to kill them is a natural phenomenon but its increase is driven by human activity^1,2^. This continual increase poses a danger to humans, with resistance already described in bacteria, virus, fungi, and parasites worldwide^3^. The World Health Organisation proposes a series of actions to tackle this issue, from preventive and educational policies to the development of strategies for immunisation, diagnosis, and treatment^4^. New drugs have rarely appeared after the Golden Age of antibiotics^5,6^, and new ones present high cost and uneven distribution between the global north and south. The high costs for drug development and actual distribution will slow innovations reaching the impoverished vulnerable areas, mostly exposed to the complications of AMR^3,6^. The development of new therapies at low cost, including traditional and non-traditional agents, is a crucial step in this battle^7^. One promising approach is the use of photodynamic therapy for antimicrobial purposes.

Photodynamic therapy (PDT) is a mode of treatment that can eliminate pathogens through oxidative stress^8,9^. The treatment process consists of a source of light, a photosensitiser (PS)—light activated compound—and the presence of oxygen. This photochemical reaction produces reactive oxygen species (ROS), leading to cellular toxicity^10^. Antimicrobial photodynamic therapy (aPDT) is an advantageous treatment as it induces non-selective oxidative damage to pathogen structures, acting on multiple targets and the likelihood of resistance development is low^8,11^. As the oxidative damage occurs in the microenvironment around the PS, the diffusion of the PS within the pathogen allows the oxidization of any structure, including the cell wall, cytoplasmic membrane, proteins, or DNA^12,13^. Therefore, aPDT has a broad spectrum of action, being able to neutralize all pathogenic agents (bacteria, fungi, protozoa, virus, and prions)^11,14^. The oxidative stress generated by aPDT could also damage the host tissues. However, mammalian cells possess a much more complex antioxidant system, with several pathways and enzymes, leading to an efficient recovery from oxidization and tolerance to the treatment^9,15^.

Current commercial PDT light sources are large and expensive machines confined to hospitals, limiting access to treatment. Alternative and cheaper light sources could make PDT more readily available, as well as allowing ambulatory treatment. Organic Light Emitting Diodes (OLEDs) are electroluminescent devices that can be produced as small, thin, flexible and lightweight wearable devices, and still provide the homogeneous and efficient light output required for ideal PDT^16^. OLED-PDT in vitro also showed efficacy of antimicrobial applications on cutaneous leishmaniasis^17^ and *Candida* strains^18^. OLEDs, either on a glass or a flexible substrate, were shown to be an appropriate light source for aPDT against bacteria, with efficient inhibition of *Staphylococcus aureus*^16^. OLED-based aPDT has also shown efficacy for inhibiting Gram-positive and Gram-negative pathogens isolated from patients with infected diabetic foot ulcers^19^. As *S. aureus* is a common commensal and potentially a dangerous pathogen, we restricted our research, on this occasion, to *S. aureus* as a model organism, with a view to study other pathogens, both Gram-negative and further Gram-positives, in the future.

Despite the advances observed in antimicrobial photodynamic therapy studies in vitro and in vivo, there is still a large gap between those studies and real-life clinical applications. The aim of our work is to show the efficacy of a novel tool to rapidly optimize antimicrobial PDT. We used a new technology called SLIC (Scattered Light Integrating Collector) which measures scattered light to measure bacterial growth with high sensitivity^20^. SLIC has been used on rapid diagnostic tests, including blood and urine samples, and for rapid antimicrobial resistance identification^21–24^. Here we show that it provides a simple way of optimising conditions for aPDT.

## Results

### OLED-based aPDT tests on SLIC

**Fig. 1:**
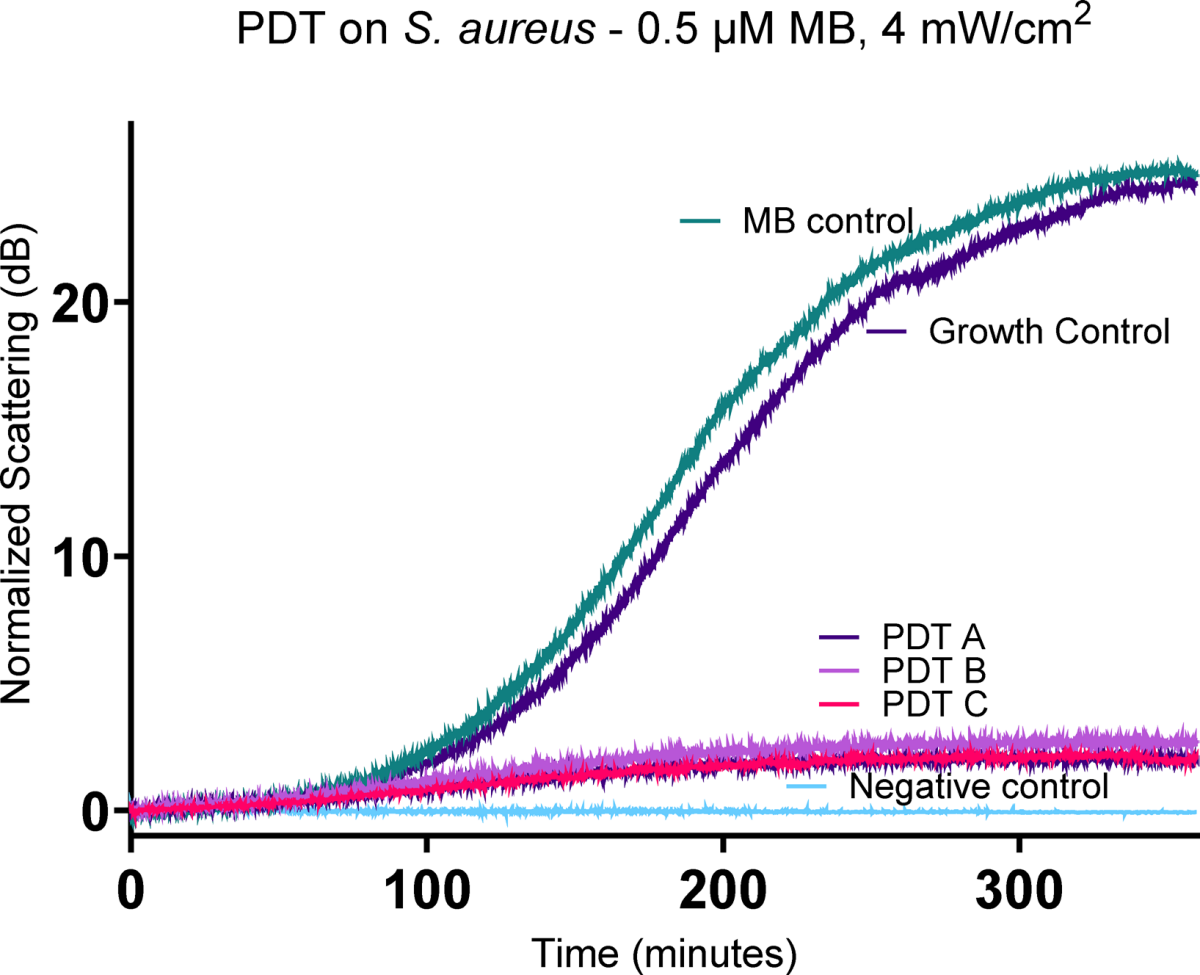
0.5 µM Methylene blue mediated PDT on *S. aureus*. *S. aureus* ~ 10^5^ CFU/ml was exposed to PDT, using 0.5 µM methylene blue and 4 mW/cm^2^, and measured on SLIC. Curves PDT A, B and C are the replicates of the treatment conditions, exposing *S. aureus* to 0.5 µM MB and light. Growth control curve is *S. aureus* on MHB2. MB control is *S. aureus* exposed to 0.5 µM MB without light. Negative control is MHB2.

Figure 1 displays the results of aPDT on *S. aureus,* using 0.5 µM of methylene blue (MB) as photosensitiser and 84.6 J/cm^2^ total energy provided by OLEDs at 4 mW/cm^2^. The graph shows normalised scattering in decibels (dB) over time (minutes). Positive control and methylene blue control show exponential growth of *S. aureus*, reaching log phase at 100 min and plateau at 24 and 25 decibels (dB), respectively, after 300 min. The treatment curves show a significant bacterial growth inhibition, with a reduction from 24.51 dB endpoint for growth control and 25.05 dB for MB control to averaged 2.28 dB for the PDT curves. The negative control shows no significant change across the whole period analysed.

**Fig. 2:**
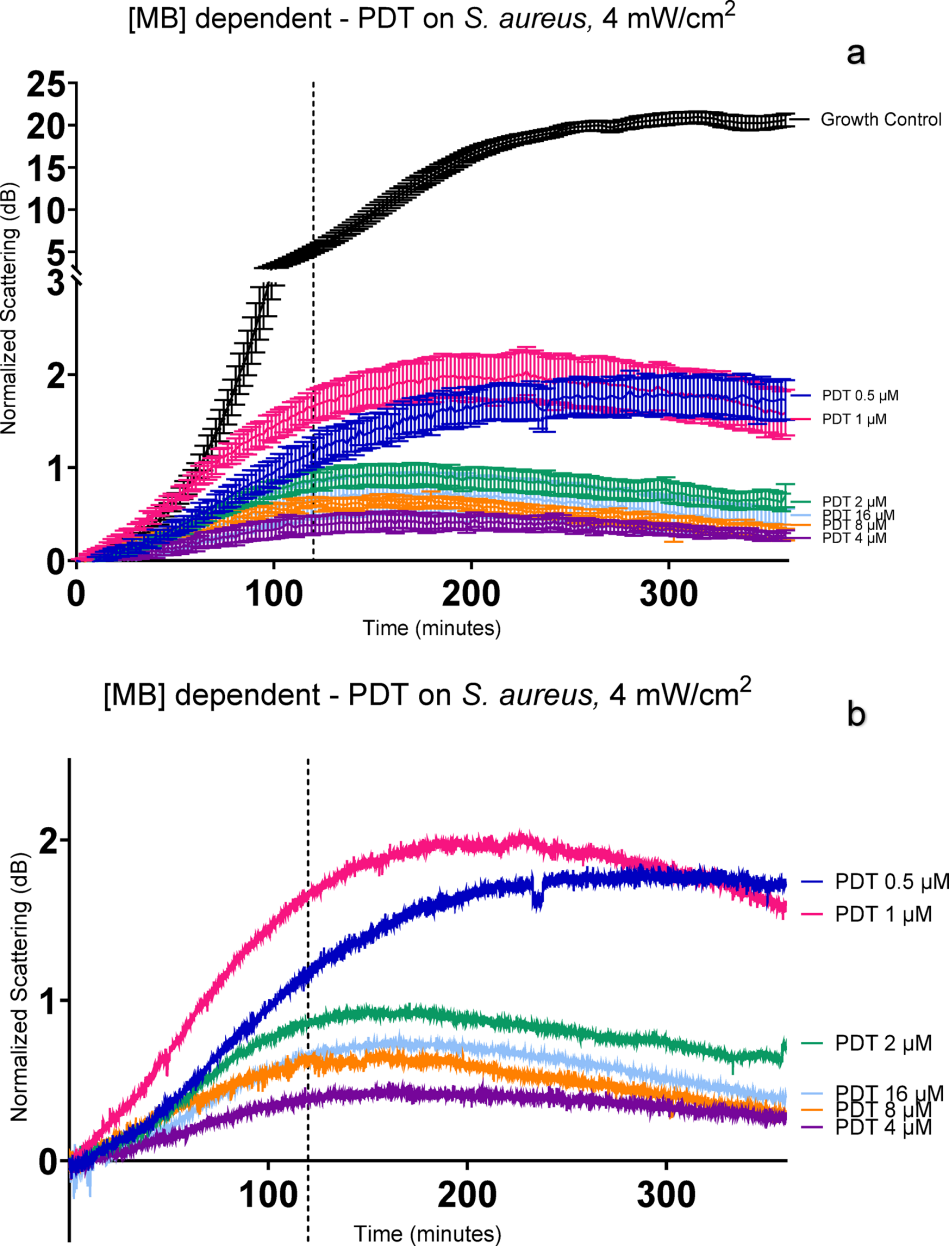
PDT effect on *S. aureus* with different concentrations of Methylene blue at 4mW/cm^2^. *S. aureus* ~ 10^5^ CFU/ml was exposed to methylene blue from 16 to 0.5 µM and4 mW/cm^2^ total light fluence (86.4 J/ cm^2^). (**a**) includes 95% confidence intervals (CI). (**b**) presents the same data with CI removed for easier visualisation.

A range of concentrations of methylene blue were analysed to evaluate the effects of increasing dosage with the same light output. Figure 2a shows the mean curves with standard error of the mean (s.e.m.) displayed as error bars and includes the growth control curve. Figure 2b shows the average of treatment curves without standard error bars to allow more detailed visualization. At the lowest concentration of MB used (0.5 μM), maximum scattering of 1.76 dB was reached at 250 min, and it maintained at that level until 360 min. Tests at 1 µM of MB showed a more steeply inclined log phase compared to the other MB concentrations, reaching exponential phase at 50 min, and a plateau at 180 min, followed by decreased scattering from 240 min. From 2 to 16 µM, the curves present a similar shape with increasing scattering until 120–140 min, reaching a peak, followed by slow decrease, with 16 µM error bars overlapping with 2, 4 and 8 µM curves (see Fig. 4 for statistical significance).

### Comparison of SLIC results to viable cells count

**Fig. 3:**
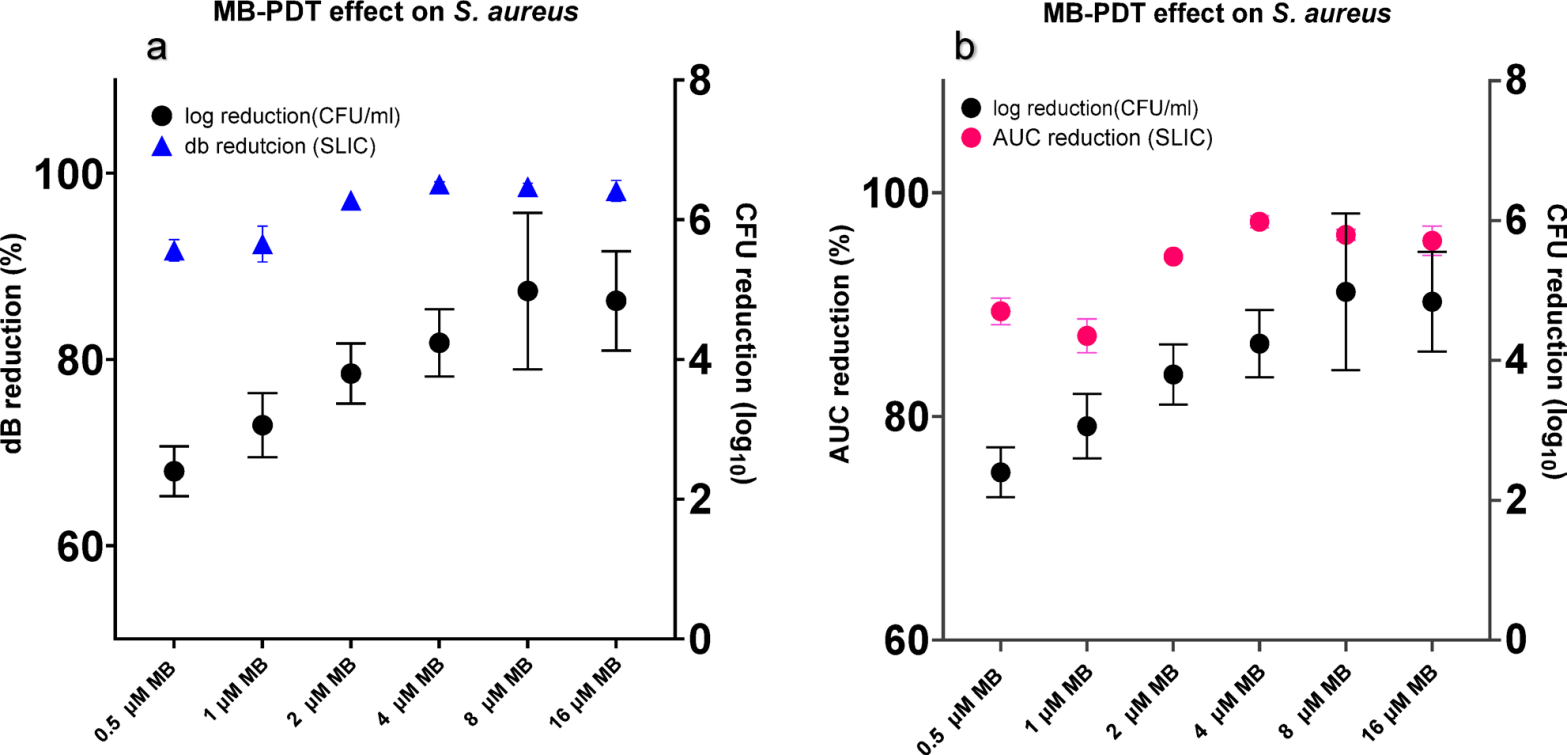
PDT effect on *S. aureus*. Reduction on bacterial load measured as log reduction (CFU count per ml) and signal reduction from SLIC. (**a**) compares log reduction to dB reduction. (**b**) compares log reduction to Area Under the Curve (AUC) reduction, Data points plotted are the mean of the values calculated for each treatment dose, and error bars represent the standard error of the mean (s.e.m.)

Figure 3 compares the results obtained from SLIC with the CFU/ml count after treatment. The log reduction represented on right Y axis on both graphs is calculated from the reduction of bacterial load as determined by CFU count after PDT, compared to the growth control. Figure 3a shows the results calculated from the end point in decibels (dB) of each treatment curve as a percentage of the endpoint of the growth control, on the left Y axis. Endpoint decibel reduction presents a similar trend to the CFU log reduction, where higher concentrations of MB induce bacterial growth suppression. Figure 3B shows the results calculated from the area under the curve (AUC) of each treatment curve as a percentage of the growth control, on the left Y axis. AUC percentage reduction for 1 µM MB was the lowest reduction observed, which is consistent with the measurement for this concentration shown in Figure 2. The highest average reduction was observed at 4 µM MB.

### Statistical analysis

**Fig. 4:**
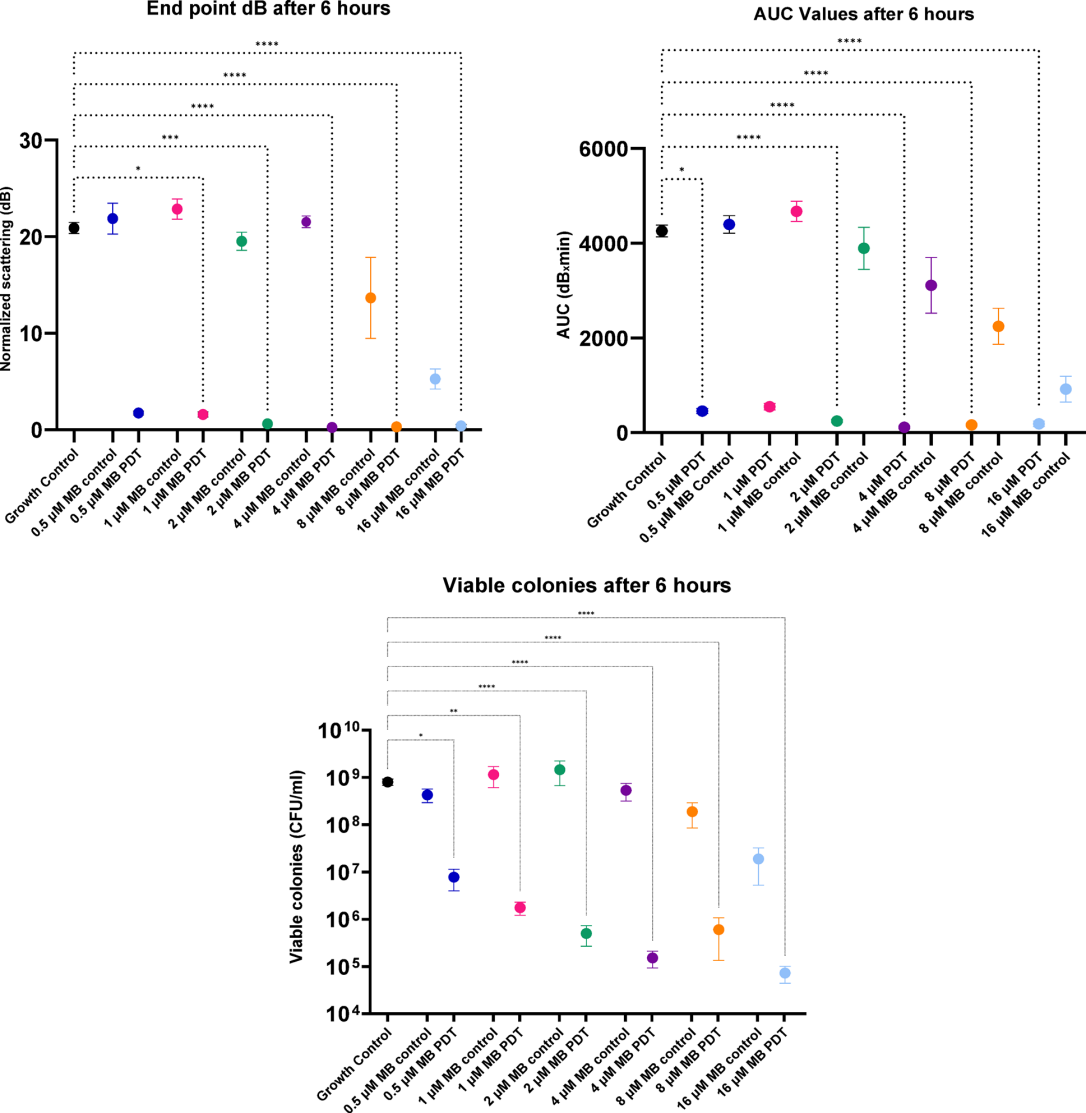
Dose dependent effect of MB and MB-PDT on *S. aureus*. Statistical analysis of PDT with different doses of MB and MB controls compared to Growth control, in dB, in AUC and in CFU/ml. Kruskal–Wallis test was performed on GraphPad Prism, adjusted *p*-value < 0.05 are marked with *. Data points plotted are the mean of the values for each treatment dose, and error bars represent the standard error (s.e.m.)

Figure 4 displays the data at 6 hours taken from Fig. 2, showing PDT results and dose dependent effect of MB Control on *S. aureus*. Data points are the mean of the values for each treatment dose, and error bars represent the standard error (s.e.m.). Different measurement metrics were applied to the data to ascertain significance, and a Kruskal–Wallis test was applied with the following results: in the case of the dB endpoint data, the PDT with 0.5 µM MB was not significantly different from the growth control (n = 9; *p*-value 0.0830); PDT 1 µM MB * (n = 9, *p*-value 0.0310); PDT 2 µM MB*** (n = 9; *p*-value 0.0002); and PDT 4 to 16 µM MB **** (n = 9; *p*-value < 0.0001) were all considered significantly different. For the AUC data, PDT 1 µM MB was not significantly different from the growth control (n = 9; *p*-value 0.0534); PDT 0.5 µM MB * (n = 9; *p*-value 0.0185); and PDT 2 to 16 µM MB **** (n = 9; *p*-value < 0.0001) were, as above, all significantly different.

For CFU/ml count data all PDT tests were significantly different from the growth control. 0.5 µM MB* (n = 9; *p*-value 0.0446); 1 µM MB ** (n = 9; *p*-value 0.0030); and 2 to 16 µM MB **** (*p*-value < 0.0001). All MB control results are not significantly different from growth controls (n = 3; *p*-value > 0.9999), in all 3 measurements (CFU/ml, endpoint dB and AUC).

## Discussion

Photodynamic therapy tests performed on SLIC allow us to analyse the dynamic change of bacterial growth over time, where increasing scattering is proportional to bacterial growth. Using SLIC, we can analyse the effects of the treatment on the dynamics of bacterial growth.

For this work, SLIC was adapted with an 820 nm laser, which is a wavelength not absorbed by methylene blue. therefore, neither absorbance nor fluorescence need be considered from the photosensitiser. In addition, due to the construction of SLIC, the photons used for treatment cannot be collected by the inbuilt photodiode and are also not to be considered on the scattering.

The evaluation of the treatment over 6 hours was initially chosen to analyse bacterial growth throughout the exponential phase, reaching stationary phase, where bacterial metabolism and replication is faster. This would provide an insight not observed before by enabling the continuous observation of the effect of aPDT on bacterial growth throughout this period. Besides, as described by Piksa et al.^14^, there is not a clear optimal radiant exposure (light fluence) as there is not enough data at low radiant exposure. The total fluence delivered here was 84.6 J/cm^2^, which falls within the effective dosage described there for all different light sources evaluated. Advancing this work includes evaluating shorter periods of irradiation.

Even the lowest concentration of MB used for aPDT presents significant growth inhibition, as observed in Fig. 1. A clear dose-dependency was not observed when evaluating the treatment with SLIC, as depicted in Fig. 2, but it is possible with SLIC to infer a threshold of the efficacy of the treatment.

The curve of 1 µM is higher than that of 0.5 µM for most of Fig. 2 (most obvious in Fig. 2b). This could be for a number of reasons including experimental variation due to bacteria in slightly different sub-stages of their phases of growth (early, mid, late logarithmic phase). The variability is included in the heights of the confidence intervals around all the traces in Fig. 2a.

When comparing the curves observed on SLIC (Fig. 2B) with the standard reduction of colony formation unit (CFU/ml) count shown in Fig. 3, curves that reach a maximal value of 1 dB are comparable to at least a 3-log reduction in cell viability. This can be observed in the tests with 2, 4, 8 and 16 μM (Fig. 2). On supplementary Fig. 1, those values are shown below 500 dB_x_min, correlating low AUC with low viable colonies.

SLIC shows a detailed plotting of how aPDT is inhibiting bacterial growth during the treatment, as we can observe the dynamic changes of scattering in real time. The curves of aPDT observed on SLIC suggest that in the initial phase (up to 140 min), some cells are still able to divide, but at a much slower rate than the growth control. This initial growth suggests that the PDT effect is limited on the doses tested. Some bacteria can still counteract the damage caused by the ROS and continue to replicate. From this point forward, the scattering starts to decrease, which suggests extensive damaged associated with bacterial cell-death. As described by Hammond et al., a decrease in scattering detection on SLIC implies that cell lysis is occurring^20^. This is also observed on Supplementary Fig. 1, with a negative AUC for data points of 16 µM of MB-PDT. A bactericidal effect is confirmed by the count of viable cells (at 16 µM of MB sub- ~ 10^5^ CFU/mL is observed).

The log variation observed suggests that at the aPDT doses tested so far, some bacteria are capable of entering a dormancy (-like) state. The antimicrobial PDT mechanism has not been fully elucidated. It is understood that ROS and oxidative stress is involved therefore it is possible that dormancy is being induced in the manner described by Peyrusson *et al*^25^. In a dormancy-like state, metabolic functions are down-regulated, meaning the effects of oxidative damage that might happen would also be decreased^26^.

The comparison of the scattering reduction with the log bacterial reduction (Fig. 3) shows that a significant log count variation is only observed between 0.5 and 1 µM MB tests, and that concentrations of 2 µM and above showed very similar log reduction. This separation into two groups of dose response suggests a threshold of treatment has been reached. In Fig. 2, the dotted line is marking 120 min, and, at this point, the efficient treatment curves are reaching the maximum values. This marks the cutline where we can define the difference between the treatment and growth control and minimize the time of the experiment to evaluate an efficient concentration of photosensitiser. As aPDT is also dependent on the light dose, the continuous exposure to light will induce a higher number of photons being absorbed by the PS overtime and conversion into ROS, increasing the effect of the treatment.

The mechanism of action of aPDT is still unclear, but using SLIC for these tests allows us to observe the microbial response in real-time. As mentioned above a 1 dB inflection from the control (growth inhibition) is directly correlated to at least a 3log_10_ reduction in viable cells. A > 3log reduction can be considered an effective antibacterial treatment^27^. SLIC is, therefore, a rapid test that can define effective treatment for novel PDT modalities in under 2 h and with real-time interrogation. This compares favourably with standard protocols which would take *at least* overnight incubation (16–24 h) to generate the same data^16,21^.

The use of SLIC allows a much faster screening of the variables that comprise aPDT, and in this case, against *Staphylococcus aureus*, a common skin commensal-come-pathogen. This, when compared to standard microbiological assays, shows that SLIC has similar potential statistical power (Fig. 4) for analytical comparison across different concentrations of treatment. With the faster screening, it is possible to more rapidly advance aPDT by screening different photosensitisers. This can be done at different concentrations, variations of light intensity and time of exposure. The tests can also be done on different bacterial and fungal pathogens and advance treatment methodologies easily and cheaply. Although we use SLIC to monitor OLED-aPDT, SLIC should be suitable for testing PDT with a range of light sources, as well as evaluating the other parameters of aPDT as described above.

Moreover, the efficacy of OLED-based aPDT shown here using low doses of aPDT (as low concentration of photosensitiser and low light fluence) can translate into a safer and more comfortable treatment for the patients when applied clinically.

In a pressing era of increasing AMR, the rapid development of new, simple, inexpensive antimicrobial therapies is crucial for adequate medical care. Our results shows that SLIC can help accelerate development of aPDT, and that OLEDs are promising for advancing this new therapy to clinical application.

## Methods

### Bacteria and culture media

*S. aureus* ATCC 25932 was resuscitated from glycerol stocks stored at − 70 °C, inoculated into 10 ml of Mueller Hinton Broth 2 (MHB2) and incubated overnight (~ 16 h) at 37 °C.

### Photosensitizer solution

The photosensitizer solution was prepared by diluting methylene blue (MB) 1.5% in filtered (syringe filter 0.22 μm) Phosphate Buffered Saline (PBS) to make 1 mM stocks and stored in the dark.

### OLED fabrication and characterization

Devices were produced as top emitting architecture with a 1.4 cm × 1.4 cm emitting area on a glass substrate by thermal evaporation (Angstrom Engineering, 03123 EVOVAC D00 vacuum deposition system) inside a glove box. The substrate was prepared by ultrasonication in acetone and isopropanol for 15 min each at 50 °C and dried with nitrogen gun. Aluminium (Al) layer 150 nm thick was used as reflective anode. The hole transport layer (HTL) was 2,2′,7,7′-tetrakis(N,N′-di-p-methylphenylamino)-9,9′-spirobifluorene (Spiro-TTB) p-doped with 2,2′- (perfluoronaphthalene-2,6-diylidene)dimalononitrile (F6-TCNNQ) (4 wt%) with a thickness of 48 nm. The electron blocking layer was 10 nm of N,N′-di(naphtalene-1-yl)-N,N′-diphenylbenzidine (NPB). The emitter layer was 36 nm of NPB as host and (2-methyldibenzo[f,h]quinoxaline)(acetylacetonate) iridium(III) (Ir(MDQ)_2_(acac)) as the emitter dopant (10 wt%). Bis-(2-methyl-8-quinolinolato)-(4-phenyl-phenolato)-aluminium (III) (BAlq) was deposited as a 10 nm thick hole blocking layer (HBL). The electron transport layer (ETL) was deposited Cesium-doped 4,7-diphenyl-1,10-phenanthroline (BPhen) with a thickness of 45 nm. A 20 nm silver (Ag) semi-transparent cathode was deposited to complete the device. Aluminium anode was deposited at 3 Å/s; Hole Transport Layer (HTL) and capping layer were deposited at 0.6 Å/s; Electron Blocking Layer (EBL), emission layer and Hole Blocking Layer (HBL) were deposited at 0.3 Å/s; Silver (Ag) cathode was at 1 Å/s. Each pixel was encapsulated individually, containing one anode and one cathode.

A Keithley 2400 was used to power the OLEDs, and their emission intensity was measured using an optometer (P9710, Gigahertz Optik). The OLEDs were driven at current densities of 1, 5, 10, 15, 20 and 25 mA/cm^2^ and their light output measured. Emission spectra were measured using an optical fibre connected to a charge-coupled device (CCD) camera of a spectrograph.

### Experimental setup of SLIC

PDT-SLIC uses an 820 nm laser for bacterial quantification. The system was adapted to contain OLEDs in the lid, with complete enclosure, working as an integrating space and is fitted with a feedback system that allows temperature control. The scattering is measured in decibels (dB) and the program HyperTerminal (hypertrum.exe) version 7 was used to log the data^28^.

### PDT tests

*S. aureus* from overnight grown cultures was diluted into MHB2 to give a starting inocula of ~ 10^5^ CFU/ml. Methylene blue was used at different concentrations (0.5, 1, 2, 4, 8 and 16 µM), diluted in PBS. PDT tests were performed in triplicates (3 biological repeats with 3 technical repeats), irradiated with the OLEDs for 6 h. The irradiance used was 4 mW/cm^2^, providing a total power of 84.6 J/cm^2^. Control samples used were methylene blue control (containing methylene blue and bacteria, without exposure to light from the OLEDs), Growth control (*S. aureus* in MHB2) and Negative control (MHB2). Measurements on SLIC were performed for 6 h at 37 °C. The viable count was determined by a modified Miles and Misra method^29^.

### Statistical analysis

For the results obtained from SLIC and plotted on Figs. 1 and 2, the initial data point is normalized as 0 dB and the following data points are compared to that. The area under the curve (AUC) was calculated based on the results obtained from SLIC and performed using GraphPad Prism version 10.0.0 for Windows (GraphPad Software, Boston, USA)^30^. Then we compared the data obtained from SLIC of the effect of OLED-based aPDT on *S. aureus.* A three-way comparison of data was then conducted consisting of; end point of dB, calculated area under the curve, and colony forming units per millilitre count (CFU/ml). A Kruskal–Wallis test was used to compare the treatment to controls (non-parametric data), also performed on GraphPad.

## Supplementary Information

Below is the link to the electronic supplementary material.

Supplementary Material 1

## Data Availability

The research data presented in this paper is available from the University of St Andrews research data repository^31^.
