# Supplementary material for: Novel optical measurement technique for antimicrobial photodynamic therapy using Scattered Light Integrating Collector (SLIC)
Source: Sci Rep. 2026 Jan 14;16:4040. doi: 10.1038/s41598-025-34122-z (PMC12855195; doi:10.1038/s41598-025-34122-z)
Supplement: Supplementary file 1 — Supplementary Material 1 [file 41598_2025_34122_MOESM1_ESM.pdf]

Supplementary Information

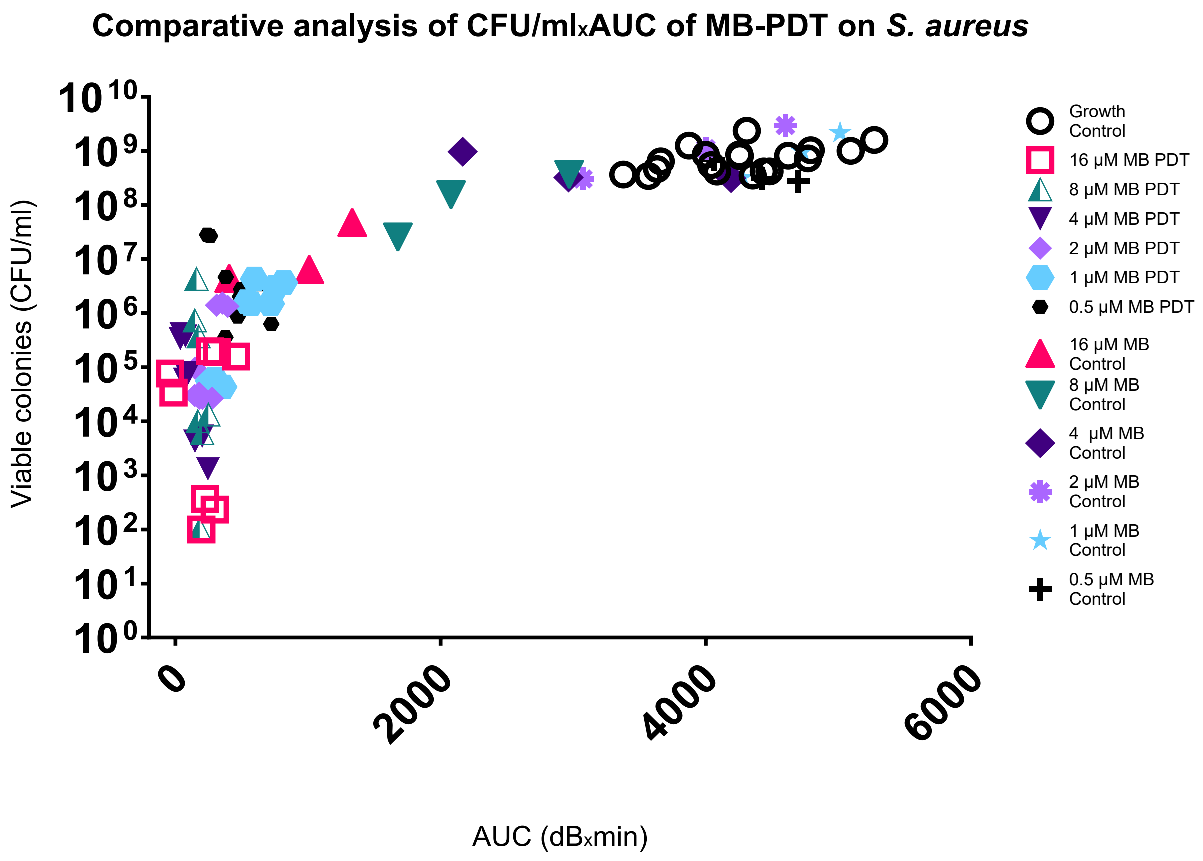

Supplementary Figure 1: **Comparative analysis of AUC versus CFU/ml as measurements of effect of different doses of MB-PDT on *S. aureus***. Each individual data point represents a single sample analysed. X axis presents the AUC calculated from the curves obtained on SLIC. Y axis shows the viable colonies count. Three data points (two 8 µM and one 16 µM MB-PDT) are not shown as the X axis is on a logarithmic scale.
